# Ferroelectric switching in ferroelastic materials with rough surfaces

**DOI:** 10.1038/s41598-019-52240-3

**Published:** 2019-11-01

**Authors:** Guangming Lu, Suzhi Li, Xiangdong Ding, Jun Sun, Ekhard K. H. Salje

**Affiliations:** 1 State Key Laboratory for Mechanical Behavior of Materials, Xi’an Jiaotong University, Xi’an, 710049 China; 2 Department of Earth Sciences, University of Cambridge, Cambridge, CB2 3EQ UK

**Keywords:** Ferroelectrics and multiferroics, Surfaces, interfaces and thin films

## Abstract

Electric switching of non-polar bulk crystals is shown to occur when domain walls are polar in ferroelastic materials and when rough surfaces with steps on an atomic scale promote domain switching. All domains emerging from surface nuclei possess polar domain walls. The progression of domains is then driven by the interaction of the electric field with the polarity of domain boundaries. In contrast, smooth surfaces with higher activation barriers prohibit effective domain nucleation. We demonstrate the existence of an electrically driven ferroelectric hysteresis loop in a non-ferroelectric, ferroelastic bulk material.

## Introduction

Ferroelastic materials are defined by their ferroelastic hysteresis^1^ in analogy with ferroelectric and ferromagnetic materials and their relevant hysteresis behavior. Additional ferroic properties often emerge when ferroelastic materials show polarity inside their domain boundaries while no such polarity exists in their bulk^2–14^. Non-polar bulk materials like SrTiO_3_ and LaAlO_3_ show strong local dipoles inside twin walls^15–20^. These polar properties do almost cancel in complex domain patterns^21^ but slight biases lead to an overall polar behavior of the material even while the bulk of the material remains strictly non-polar^22^. Electric fields may hence influence such domain structures^23,24^ and the question arises whether hysteretic behavior itself can be induced by electric fields. This would mean that ferroelastic materials are indeed also (weakly) ferroelectric due to polar domain boundaries. The same question arises for ferromagnetic domain walls, namely whether magnetic fields can switch ferroelastic materials if domain walls contain ferromagnetic properties? A simple answer is found when we consider simple ferroelastic needle domains. These domains can be propagated and retracted by electric fields, which constitutes ferroelectricity^25^ although the ferroelectric, switchable polarization is very small. In addition, an electric field can switch the spontaneous strain so that a ferroelastic/ferroelectric hysteresis is observed.

A more stringent test is the switchability of uniform samples. In this case, the switching needs an additional, less obvious mechanism: domains with different elements of the strain tensor need to nucleate in order to switch domains. Here we focus on the nucleation and growth of ferroelastic domains under an applied electric field. In order to explore this effect we follow previous work and simulate the switching by molecular dynamics techniques^26^. We distinguish the current models according to their treatment of free surfaces. We take into account a single ferroelastic domain with two kinds of surfaces, namely (i) a charge-free smooth surface with no excess net charges at surface layers with an equal number of A and B atoms^25–27^ and (ii) rough surface with excess net charges at surface steps. Electric fields are then applied to study the evolution of domain patterns and generate a polarization hysteresis loop.

## Results

**Figure 1:**
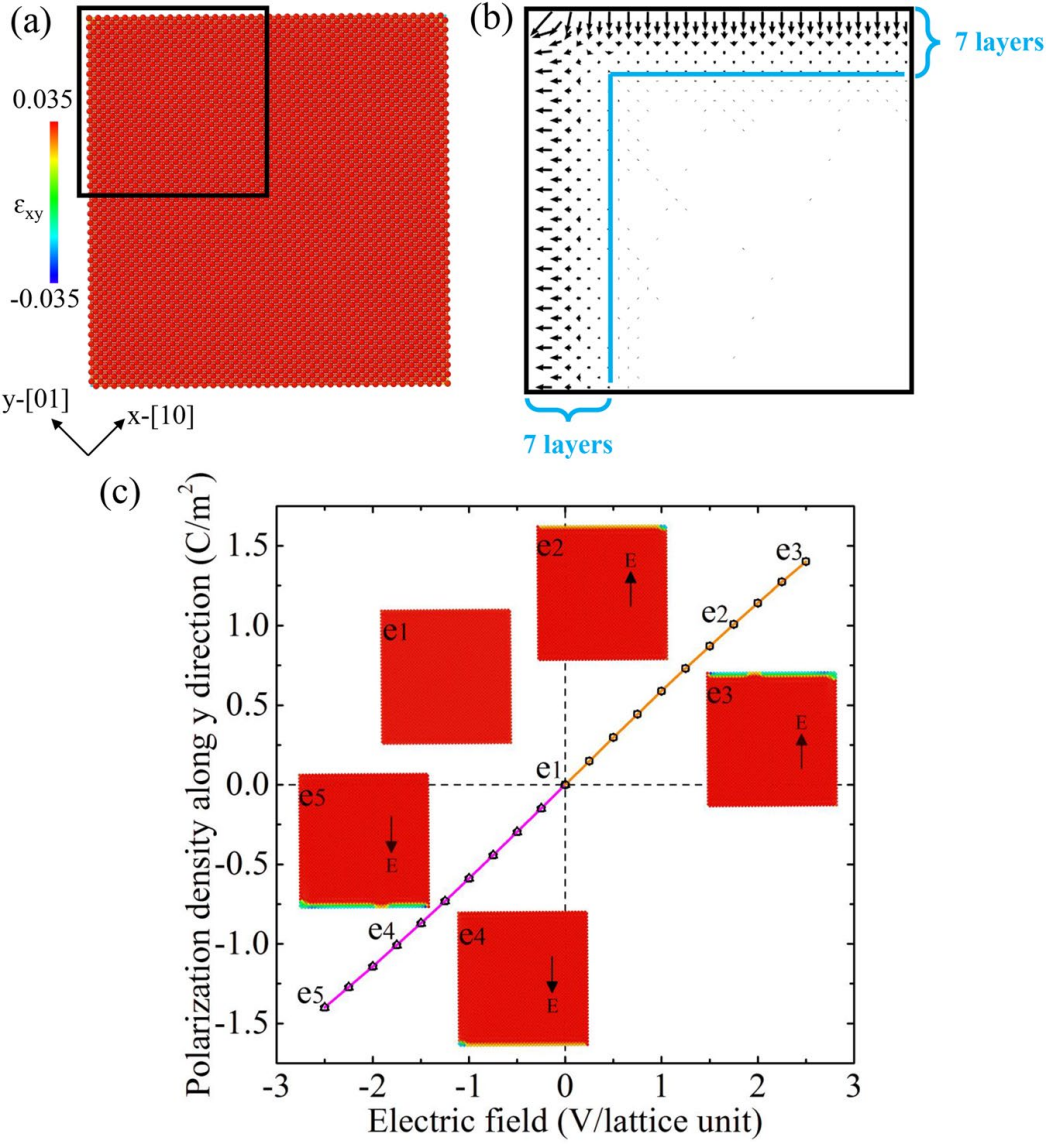
(**a**) Fully relaxed single domain configuration with smooth charge-free surfaces. (**b**) The dipole configuration near the surfaces in the area indicated by the black frame in (**a**). Dipole displacements are amplified by a factor of 200 for clarity. (**c**) The variation of polarization density along y direction (P_y_) as a function of external electric field applied along the [11] direction with smooth surfaces. Virtually zero hysteresis is observed during the field cycle. The insets show the typical microstructures at different states as indicated in e1-e5. The black arrows in e1-e5 indicate the direction of the external electric field. The colors in the atomic images are coded by the atomic-level shear strain (ɛ_xy_).

We start from a single domain configuration with {11}-type smooth surfaces, which is cut from a bulk containing A and B sublattices with orientation of *x*-[10] and *y*-[01], as shown in Fig. 1(a). The free surfaces are charge-free with an equal number of A and B atoms in the surface layer. The dipoles are induced by the surface relaxation in the surface layers with around ~7 lattice unit thickness. The dipoles are perpendicular to their respective surfaces and disappear in the inner part of the sample due to the inversion symmetry of the bulk material (see Fig. 1(b)). The orientations of the surface dipoles on the left surface point outward while pointing inward on the nearby top surface, which differs from the unsheared ‘cubic’ structure from where all surface dipoles point inward (see Fig. S1 in Supplementary Material).

We first explore the domain nucleation at smooth surfaces by an electric field, which is applied along the [11] direction (upwards) and retraced back (downwards). Figure 1(c) shows the variation of macroscopic polarization density along *y* direction (P_y_) under the external field. The change of polarization density along the *x* axis (P_x_) behaves similarly to that of P_y_. The insets show the typical snapshots at different states as marked in e1-e5. As the electric field increases, the new domains nucleate near the corner (e2) and propagate by kinks movement (e3). The domain switching is only limited to a very thin layer (~3 lattice units) near the surface. When the electric field is retraced, the new domain switches back. Similar surface nucleation (e4) and kink movements (e5) are observed near another surface as the field is applied in the opposite way ([-1-1] direction). The polarization hysteresis loop is hardly observable in Fig. 1(c). The driving forces for domain nucleation in the corners stems from the large coupled strains induced by these corners under the applied electric field.

**Figure 2:**
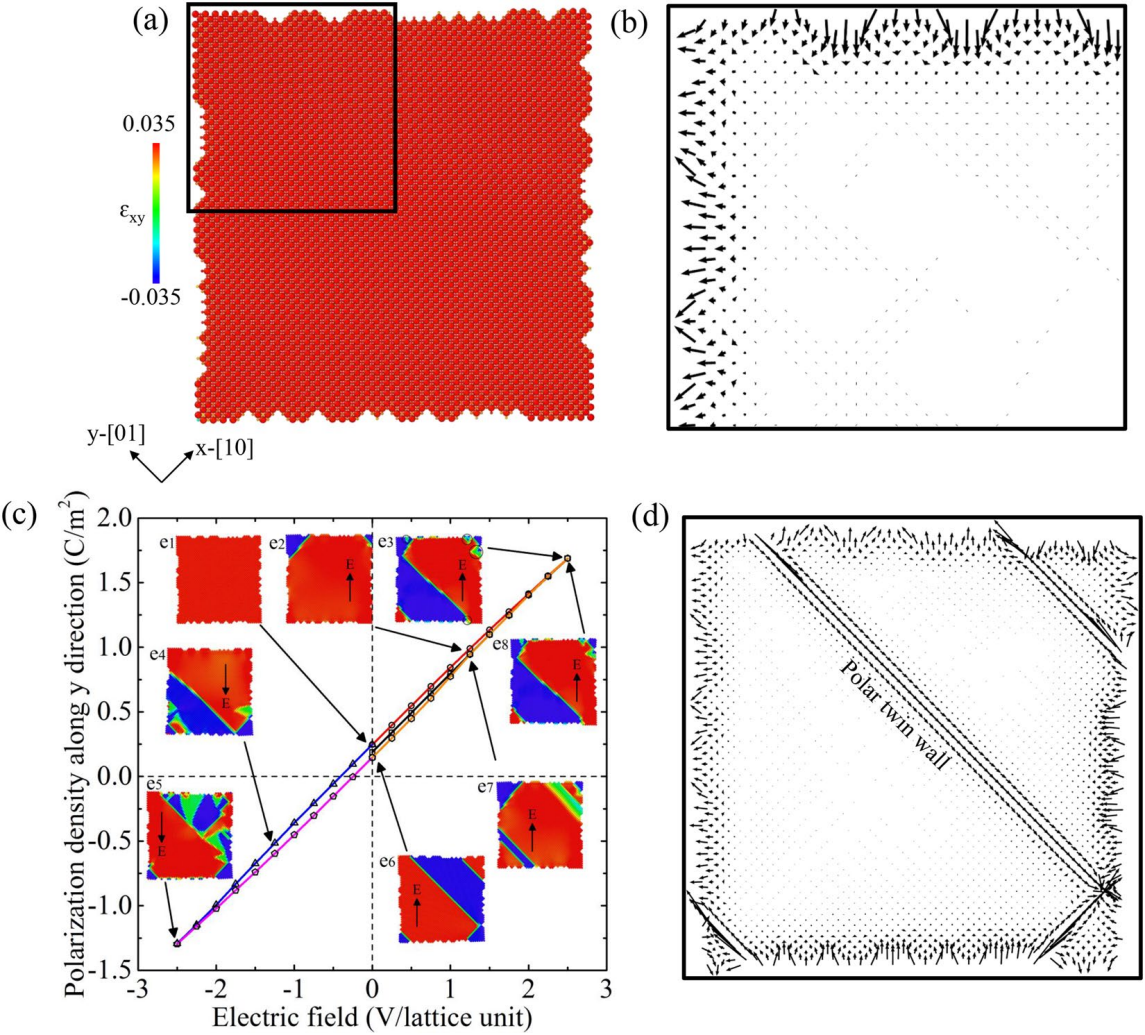
(**a**) Fully relaxed single domain configuration with rough surfaces (containing locally nanoscale steps). (**b**) The dipole configuration near surfaces in the area indicated by the black frame in (**a**). (**c**) The variation of polarization density along y direction (P_y_) as function of the external electric field along the [11] direction with rough surfaces. A small ferroelectric hysteresis was observed during field cycling. The insets show typical microstructures at different states as shown in (e1)-(e8). The black dashed arrows in (e1)-(e8) indicate the direction of the external electric field. The colors in the atomic images are coded by the atomic-level shear strain (ɛ_xy_). (**d**) The dipole configuration at state e6 in (**c**). The nucleated domains are polarized near the domain boundary. Dipole displacements are amplified by a factor of 200 for clarity.

We now explore the influence of surface roughness on domain nucleation. The rough surface was constructed with randomly distributed steps. These steps have net charge due to the breaking of AB stacking at surfaces. Figure 2(a) shows the configuration of single domain with nano-scale steps at free surfaces. These steps contain charges and are similar to classic charged defects (vacancies, holes etc.) seen in many ferroic materials^28^. Figure 2(b) shows the dipole configuration in the local area near the free surface. The surface roughness adds local “noise”,and polarization, which is stronger at rough surfaces than at smooth surfaces (Fig. 1(b)). The thicknesses of the polarized surface areas in the two systems are basically the same (~7 lattice units). These locally surface steps break the macroscopic inversion symmetry, producing a net polarization density even without electric field as P_x_ = −0.373 C/m^2^ and P_y_ = 0.187 C/m^2^.

We then apply the electric field in a [11] (upwards)/[-1-1] (downwards) cycle. Figure 2(c) shows the variation of polarization density along y direction (P_y_) as function of the external field. We observe a typical ferroelectric hysteresis loop during field cycling. The insets illustrate the configuration of domain patterns at different states as indicated by e1-e8. Starting from a single domain with a very small net polarization (e1), the new domains initially nucleate at surface steps and corners as the electric field increases (e2). The movement of the twin walls then progresses by nucleation and propagation of kinks (e3). Besides the two-oriented stable domains (red and blue areas), we also find that some areas near the surface stay in the intermediate state after the transition (green areas in e3). Reducing the field leads to domain back-switching. When the field is further applied in the [-1-1] direction (downwards), a similar process of domain nucleation and propagation is observed (e4-e5). The switching is reversible and reproducible during the hysteresis loop (e6-e8). Since the switchable polarity is only generated from free surfaces and domain boundaries (see Fig. 2(d)), the ferroelectric hysteresis loops are very thin. Nevertheless, they are sufficient to generate ferroelectricity while the bulk material remains centro-symmetric and purely ferroelastic.

**Figure 3:**
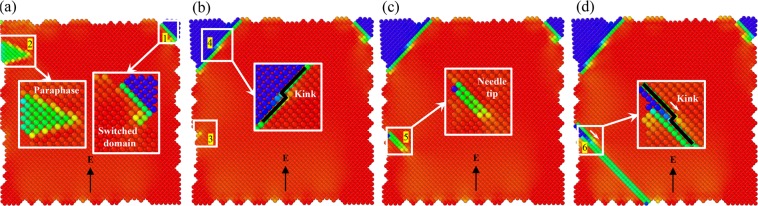
The evolution of domain pattern driven by the external electric field. (**a**) Nucleation of new domains. Site 1 shows the switched domains. Site 2 shows the intermediate state during transition. (**b**) Growth of new domain and nucleation of needle domain. Site 3 shows the transient nucleation state of needle domain from the rough surfaces. Site 4 shows the stable new domain after switching. (**c**) The propagation of needle domains nucleated from site 3. (**d**) The propagation of domain wall via the moving kink, as marked in site 6.

Figure 3 shows the surface nucleation and wall movements in ferroelastic domain switching. The initial domain nucleation occurs near the corner and steps at the left surface. We observe the formation of stable new domain (marked ‘1’ in Fig. 3(a)) and also unstable patches in the transition state (marked ‘2’ in Fig. 3(a)). As the field increases further, these unstable patches switch to the new domain (marked ‘4’ in Fig. 3(b)). Simultaneously, the needle domain nucleated from the step sites with relatively low energy barrier (marked ‘3’ in Fig. 3(b)). Domain switching progresses by the nucleation and growth of needle domains mediated with the kink mechanism (marked ‘5’ in Fig. 3(c)). Driven by the electric field, the rough surfaces emit kinks and the new domains grow by kink propagation (marked ‘6’ in Fig. 3(d)).

**Figure 4:**
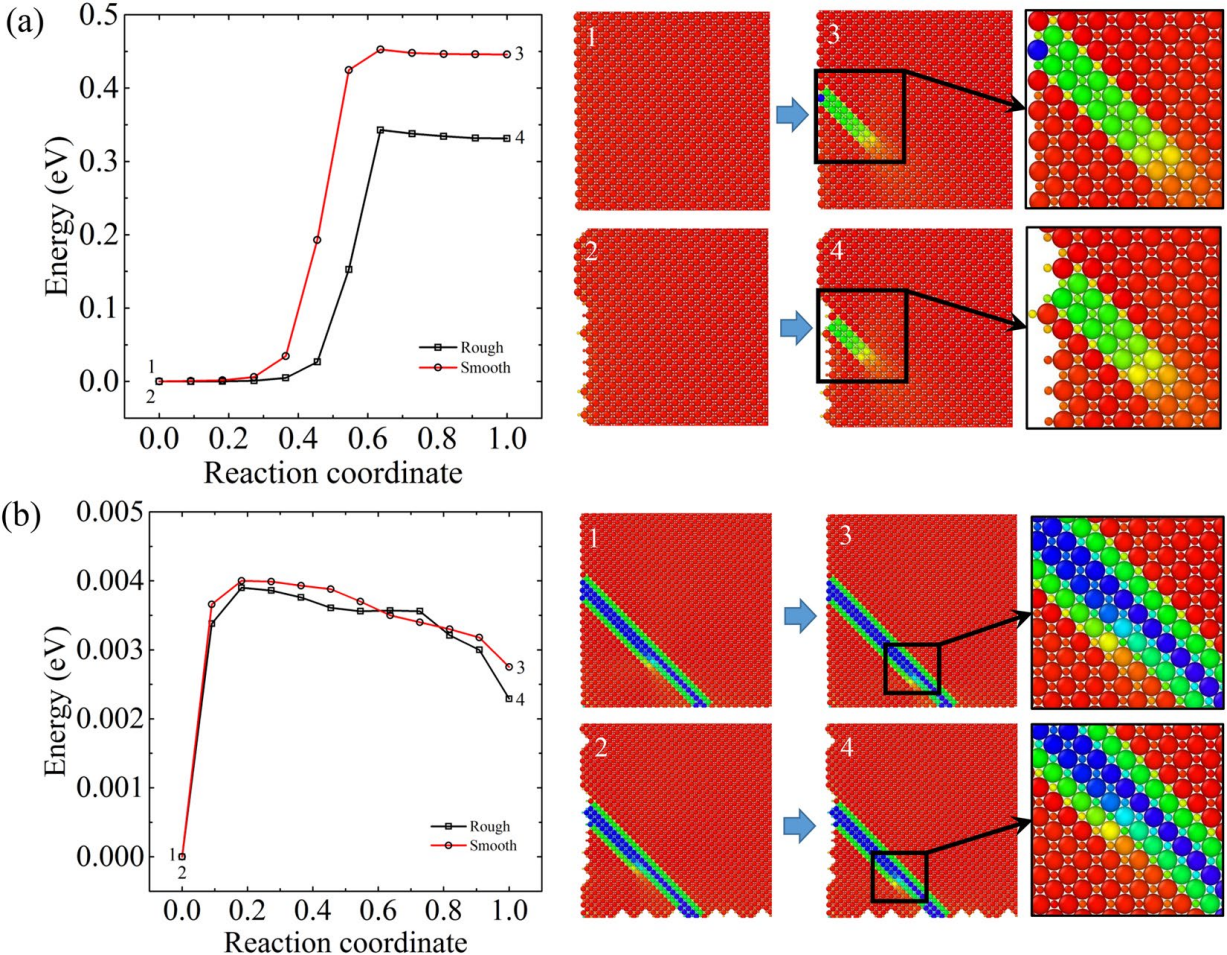
The activation barrier for (**a**) domain nucleation and (**b**) kink propagation in systems with smooth and rough surfaces. The atomic images are coded with the atomic-level shear strain (ɛ_xy_).

Our results show that it is much easier to initiate domain nucleation at rough surfaces compared with smooth surfaces. The corners and steps in the rough surface are the primary nucleation sites. We calculated the activation barrier for domain nucleation by using the climbing image nudged elastic band (CINEB) method^29^. Figure 4(a) shows the optimized path for nucleation at smooth surface and surface steps. The nucleation at smooth surfaces needs to overcome a barrier of ~0.45 eV. This barrier is reduced to ~0.35 eV once the surface contains steps. The surface roughness effectively reduces the barriers for domain nucleation at free surfaces driven by external electric fields, as that shown in ferroelectrics^30^. We also calculate the barrier for kink propagation inside twin boundaries. These barriers are very weak (~0.004 eV) and are identical for both systems (Fig. 4(b)). Thus, once a kink is nucleated, it is very easy to move the kink to facilitate domain switching under the external electric field.

## Discussion

The observation of an electric hysteresis in purely ferroelastic materials is related to the polarization of domain walls and is greatly facilitated by rough sample surfaces. The movement of ferroelastic domains in materials like SrTiO_3_ is further enhanced by dielectric pressure because the dielectric parameters are highly anisotropic and favor or disfavor specific domain orientations^24^. In our model, the polarization is entirely localized inside domain walls and near the rough sample surfaces, which agrees with earlier results by phase field modelling^31^. The applied electric field rotates the polarization and stabilizes (or destabilizes) domain walls because their polarization vector is no longer compatible with the adjacent bulk material. The rough surfaces carry relatively large and randomly distributed polar vectors. The (de-) stabilization effect is much stronger than that for smooth surfaces where all surface dipoles are orientated perpendicular to the surfaces and only the corners act as nucleation sites. The polarization hysteresis is hence a result of the nucleation of interfaces near surfaces and the subsequent evolution of domain pattern. The magnitude of polarization strongly depends on the twin boundary density. In realistic cases, the concentration of interfacial atoms is rather small, namely in the order of 1 ppm (part per million). This explains why the ‘parasitic’ hysteresis is very slim. Nevertheless, we have shown that interfacial ferroelectricity is a physically viable proposition that explains previous observations^32–35^.

## Methods

The molecular dynamics simulations are implemented based on a two-dimensional toy model consisting of two base charged atoms (A and B)^21,36^. The twin structure is constructed by the anion sublattice A whose interaction contain anharmonic elastic interactions (Landau springs) while the interactions between atoms of the B sublattice and between A and B atoms are purely harmonic. Flexoelectricity is caused by strain gradients and is therefore restricted to generate polarity near twin walls and surfaces^21^. The parameters for A-A and B-B interactions are illustrated in our previous paper^21^. However, the coupling coefficient between A and B sublattices is modified to be stronger (${V}_{{\rm{AB}}}={\rm{3.5}}{({\rm{r}}-\frac{\sqrt{2}}{2})}^{2}$) in order to strengthen the coupling effect between strains (ɛ) and charges (q* = *1.60 × 10^−19^ C)^15,18,37^. The model parameters and potential form are summarized in Table I in the Supplementary Materials. The dielectric constant is chosen to be isotropic, and set to 1000 in order to avoid the additional effect of dielectric pressure on the domain wall movements. The boundary condition is ‘open’ (free boundary condition) in both two dimensions, i.e. the surface atoms are specified to be stress-free (Neumann condition). We construct two kinds of surfaces. (i) The surface is smooth containing the same number of A and B atoms. Such smooth surface has no excess charge except at four corners. (ii) Roughness is added at the surface. The depth of surface steps is around three atomic distances. These steps contain excess charges because the charge balance between A and B atoms is not conserved locally (but globally where we have an equal number of A and B atoms). Our more extended simulations were conducted at low temperature at T = 0.001 K to avoid thermal effects^38,39^. The electric field is applied stepwise along the [11] direction. For each step, the system relaxes for 100 ps and the snapshots within the last 50 ps were averaged to show the microstructure and the polarization. All calculations were carried out by using the LAMMPS code^40^.

## Supplementary information

Supplementary information

## Data Availability

All data supporting the results of this study are available within the paper and Methods.
