# Supplementary material for: Ferroelectric switching in ferroelastic materials with rough surfaces
Source: Sci Rep. 2019 Nov 1;9:15834. doi: 10.1038/s41598-019-52240-3 (PMC6825142; doi:10.1038/s41598-019-52240-3)
Supplement: Supplementary file 1 — Supplementary information [file 41598_2019_52240_MOESM1_ESM.pdf]

Supplementary Information

Ferroelectric switching in ferroelastic materials with rough surfaces

Guangming Lu[1,2], Suzhi Li[1*], Xiangdong Ding[1*], Jun Sun[1] and Ekhard K. H. Salje[1,2*]

1 *State Key Laboratory for Mechanical Behavior of Materials, Xi'an Jiaotong University, Xi'an 710049, China*

2 *Department of Earth Sciences, University of Cambridge, Cambridge CB2 3EQ, UK*

Corresponding author: lisuzhi@xjtu.edu.cn; dingxd@mail.xjtu.edu.cn; ekhard@esc.cam.ac.uk

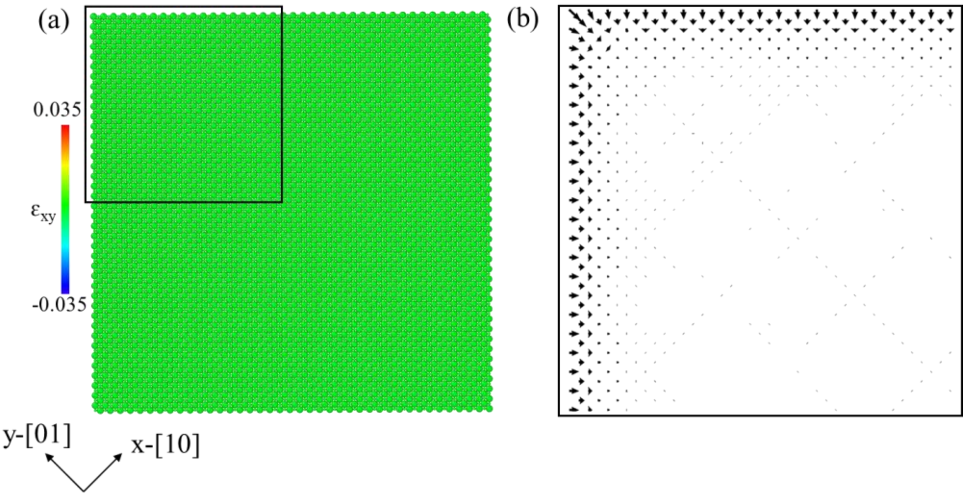

Fig. S1 (a) Fully relaxed unsheared 'cubic' configuration with smooth charge-free surfaces. (b) The dipole configuration near the surfaces in the area indicated by the black frame in (a). Dipole displacements are amplified by a factor of 200 for clarity.

Table I. The interaction potential in binary system. For different ranges of nearest neighbor (NN) interactions, we set different forms of potential function to describe them. Besides the short-range interactions, the long-range Coulomb forces were considered. The dielectric constant for Coulomb interaction is 1000.

Interactions	Short range	Potential form
A-A	First NN	$20(r-1)^2$
	Second NN	$-10(r-\sqrt{2})^2+8000(r-\sqrt{2})^4$
	Third NN	$8(r-3)^4$
	Fourth NN	$-10(r-\sqrt{5})^2+5100(r-\sqrt{5})^4$
B-B	First NN	$20(r-1)^2$
	Second NN	$1.5(r-\sqrt{2})^2$
A-B	First NN	$3.5(r-1)^2$
